# Risk and prognostic factors of survival for patients with pancreatic ductal adenocarcinoma metastasis to lung: a cohort study

**DOI:** 10.3389/fonc.2025.1521616

**Published:** 2025-05-21

**Authors:** Yi Chen, Xingyu Liu, Jun Yu, Jianshui Li, Jingdong Li, Pengsheng Yi, Bin Wu, Guangnian Zhang, Dawei Deng, Yong Li, Shu Yan, Lin Ma, Chuan Lan

**Affiliations:** ^1^ Department of Hepatobiliary and Pancreatic Surgery, The Affiliated Hospital of North Sichuan Medical College, Nanchong, China; ^2^ Department of Clinical Medicine, North Sichuan Medical College, Nanchong, China

**Keywords:** pancreatic ductal adenocarcinoma, lung metastasis, predictors, Surveillance Epidemiology and End Results (SEER) database, logistic regression, nomogram

## Abstract

**Background:**

Currently, there is no validated model for predicting the occurrence and prognosis of lung metastases (LM) in patients with pancreatic ductal adenocarcinoma (PDAC). We aimed to construct a nomogram for risk prediction and a prognostic model to guide clinical practice.

**Methods:**

In total, 10,813 patients were enrolled from the Surveillance, Epidemiology, and End Results (SEER) database between 2010 and 2015 and divided into training and internal validation cohorts at a ratio of 7:3. Following nomogram construction, data of patients diagnosed with PDAC were retrospectively collected for external validation.

**Results:**

Using multivariate logistic regression analysis, larger tumour size, primary tumour site in the body or tail of the pancreas, bone metastasis, and liver metastasis were associated with LM. Furthermore, through multivariate Cox analysis, we found that LM was associated with a poor prognosis in patients with PDAC. Patients who underwent surgery or chemotherapy had better prognoses.

**Conclusion:**

The two nomograms showed excellent performance in the training and internal validation cohorts and a favourable performance in the external validation. The prognostic nomogram divided the patients into high- and low-risk groups based on mortality. The LM risk and prognostic prediction model in PDAC showed high accuracy and reliable clinical application.

## Introduction

Globally, pancreatic ductal adenocarcinoma (PDAC) stands as the foremost reason for mortality associated with cancer, surpassing breast cancer as the third leading cause of cancer-related deaths in the United States (US) and is forcasted to be the second leading cause of cancer-related deaths in the US by 2030 (1, 2). In China, PDAC ranks ninth among the frequently encountered malignant tumours and the sixth prevalent cause of mortality (3). PDAC is characterised by a grim prognosis and high lethality, exemplified by a 5-year relative survival rate of roughly 10% in the US (4). Many factors affect the prognosis of patients with PDAC, of which distant metastasis is a vital factor. At the time of diagnosis, the majority of patients are found to have either locally advanced (30-35%) or metastatic (50-55%) stages of the disease (5).

In PDAC, the liver is the predominant site for distant metastasis, with the lung being the subsequent most frequent location (referred to as lung metastasis, or LM). The incidence of LM occurring after the initial diagnosis or recurrence of pancreatic cancer (PC) has been reported to be 4.76%–12.3% (6, 7). Three pathways are involved in developing LM: lymphatic metastasis, blood transport metastasis, and direct infiltration. Mechanistically, the metastatic process includes alterations in cancer cell surface adhesion molecules, transmembrane signal distortion, genetic changes, tumour immune microenvironment changes, and exosomes (8). The National Comprehensive Cancer Network (NCCN) guidelines define LM as stage IV, with an extremely poor prognosis. For patients diagnosed with isolated lung metastasis (LM), the reported median Overall Survival (OS) was 561 days, with a median Recurrence-Free Survival (RFS) of 748 days, and a median Progression-Free Survival (PFS) of 307 days (9). Compared with patients with localised tumours, the 5-year survival rate of patients with metastatic PDAC decreases significantly from 42% to 3% (10). Therefore, early diagnosis and detection of PDAC in patients with LM are essential.

Imaging examination is the preferred option for the preliminary assessment of metastasis in clinical scenarios, including computed tomography (CT), magnetic resonance imaging (MRI), and fluorodeoxyglucose positron emission tomography/computed tomography (FDG-PET/CT); however, each has its advantages, with no way to determine which has the best evaluation ability (11–15). The detection efficiency may be improved with a combination of these methods. Tissue biopsy is the gold standard for diagnosing metastatic carcinoma but is not routinely used for early diagnosis unless necessary. Liquid biopsy has attracted wide attention due to the minimal associated trauma and its ability to assess tumour heterogeneity; however, it has no standardised detection method and is costly; thus, it is not used in the clinic (16, 17). Accordingly, we urgently need to establish a new practical clinical diagnosis modality and improve the diagnostic efficiency of patients with PDAC and LM to improve prognosis.

Conventional clinical treatments for PDAC include surgery, chemotherapy, and radiotherapy. The PDAC diagnosis and treatment guidelines do not recommend surgical resection for patients with distant metastases. However, in a national multicentre study, Japanese scholars showed that surgery is feasible for PDAC with isolated pulmonary metastases and can be supplemented with gemcitabine (18). In the absence of contraindications, chemotherapy is the routine treatment after radical resection of PDAC, and the separate use of gemcitabine or fluorouracil is recommended (19). However, PDAC shows some resistance to chemotherapeutic drugs, especially gemcitabine. Studies have shown that PDAC cells are more resistant to gemcitabine than other chemotherapeutic drugs (20, 21). This greatly limits the use of gemcitabine and reduces the patient’s prognosis. Therefore, there is an urgent need for practical tools and useful clinical treatment options for PDAC with distant metastases.

The Surveillance, Epidemiology, and End Results (SEER) program gathers demographic, clinical, and outcome information for all cancer diagnoses within selected geographical areas and subgroups across the United States, covering approximately 48% of the total number of patients with cancer in the US population (22). As visual tools, nomograms are widely used in oncology research to build diagnostic and prognostic cancer models. One of the main advantages is the ability to individualise risk estimation based on patient and disease characteristics, which is superior to clinicians’ judgment regarding disease progression (23, 24). Furthermore, due to its significantly better clinical efficacy, the nomogram has replaced the tumour–lymph node–metastasis (TNM) staging system as a new standard for tumour diagnosis (25, 26). Nevertheless, no relevant nomogram has been established for the diagnostic prediction of PC with LM (PCLM). Therefore, the objective of this research was to develop and verify an effective nomogram for the clinical diagnostic prediction of PCLM.

## Methods

### Patient selection

We extracted data from patients newly diagnosed with PDAC between 2010 and 2015 from the SEER database. The following inclusion criteria were applied: (1) complete survival and follow-up data; (2) PC as the primary tumour (topographical codes from the International Classification of Diseases for Oncology ICD-O-3: C25.0-C25.3, C25.7-C25.9); (3) histological subtype PDAC (ICD-O: 8140, 8480, 8500); and (4) a clear pathological diagnosis of PC and an imaging diagnosis of LM.

The exclusion criteria were as follows: (1) incomplete baseline information, such as age, sex, race, grade, stage, treatment, and metastasis, and (2) patients with unknown primary tumours and metastatic status. Finally, our study included 10,813 patients diagnosed with PDAC, including 445 patients with LM.

**Figure 1:**
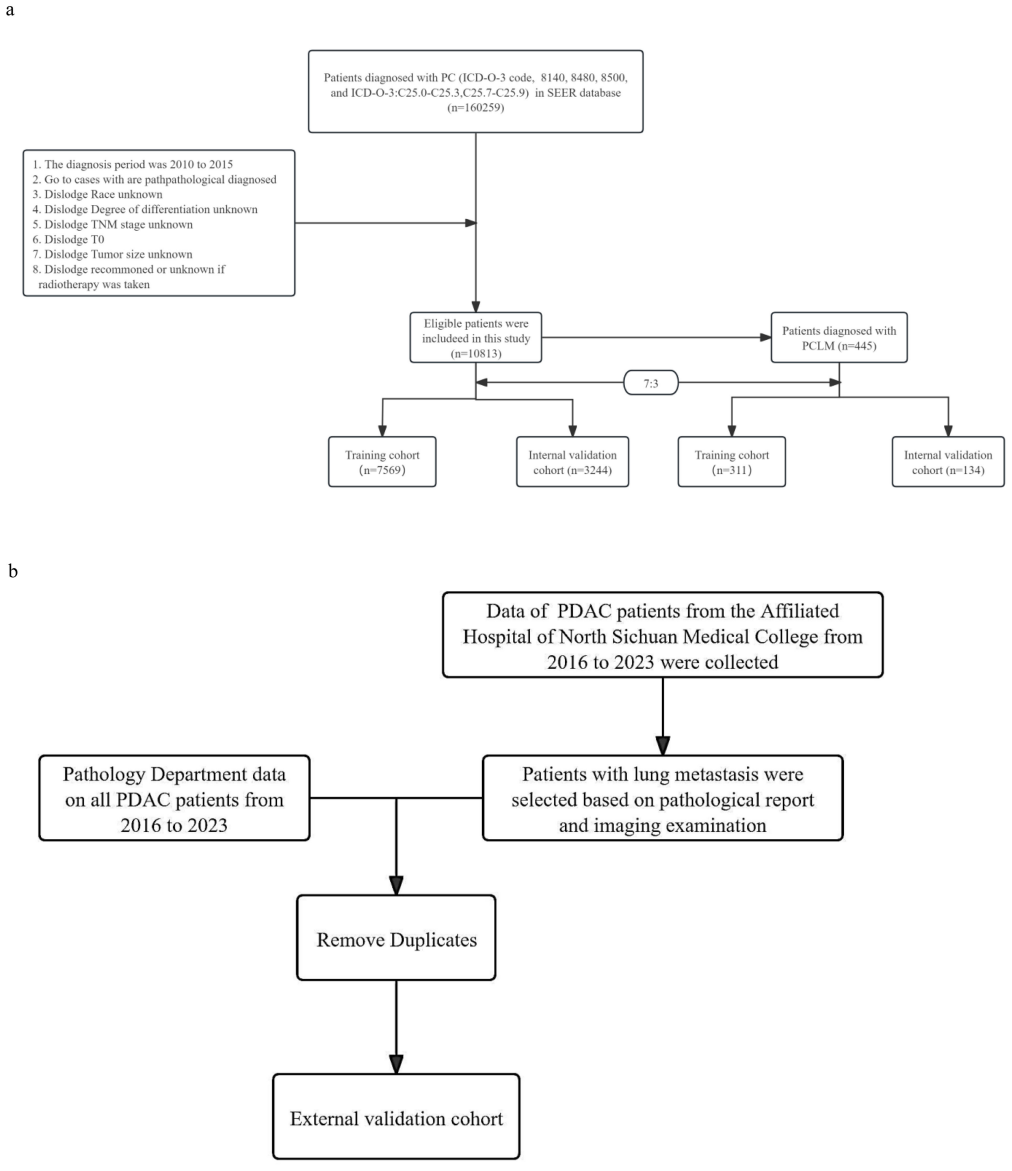
**(a)** Flow chart of patient inclusion and grouping. **(b)** Flow chart of patient selection in the external validation cohort.

In addition, we retrospectively collected data from patients with PDAC and LM at the Affiliated Hospital of North Sichuan Medical College between 2016 and 2023 as an external validation cohort. The inclusion and exclusion criteria for the external validation cohort were consistent with those of the internal cohort. The flow chart for patient selection is shown in **Figures 1a, b**. The research program was approved by the Institutional Review Committee of the Afemittee Hospital of North Sichuan Medical College (number: 2024ER568-1), and the requirement for informed consent was waived because it’s a retrospective study. Otherwise, all methods were performed in accordance with the relevant guidelines and regulations.

### Baseline characteristics

Baseline characteristic data were used to analyse the risk factors of LM in PDAC, including age at diagnosis, race, sex, grade, primary site, American Joint Committee on Cancer (AJCC) T stage, AJCC N stage, tumour size, and metastasis status. The following variables were collected to assess the prognosis of patients with PDAC with LM: age, sex, race, income, marital status, grade, tumour size, primary site, AJCC T stage, AJCC N stage, delayed treatment, surgery (performed or not performed), radiation (performed or not performed), chemotherapy (performed or not performed), and metastasis status. The optimal tumour size cut-off was determined by x-tiles software, and the subjects were divided into three groups according to the tumour size: ≤25 mm, 26–40 mm, and ≥41 mm (27). Finally, we used an electronic medical record system to collect the baseline characteristics of patients with PC in the external validation cohort.

### Statistical analysis

First, patients were randomly divided into training and internal validation cohorts (7:3). Chi square and Fisher’s exact tests showed no significant differences in variables between cohorts (P > 0.05). Univariate logistic analysis was done first. Variables with P < 0.05 were selected for multivariable logistic analysis to determine LM risk factors in PDAC. Univariate Cox regression was used for prognostic factor analysis (P < 0.05), and these variables were included in multivariate Cox regression to determine independent prognostic factors for PDAC with LM. Odds ratios (ORs) and 95% confidence intervals (95% CIs) were calculated. Then, two nomograms based on risk and prognostic factors were established via the “rms” package in R. Receiver operating characteristic (ROC) curves were used to assess nomogram accuracy, and decision curve analysis (DCA) was used to test clinical utility (28, 29). Calibration plots were used to estimate prediction actual observation consistency. DCA was used to quantify net gain at different threshold probabilities to compare nomograms and other models (30). We verified the nomogram’s clinical utility by comparing ROC and DCA curve areas of the nomogram and TNM stages in training and validation cohorts. The prognostic nomogram’s predictive value was verified using high and low risk survival curves based on median risk score (31). In this study, overall survival (OS) from diagnosis to death (due to cancer) was the prognostic survival outcome. Finally, an external validation cohort was used to evaluate efficacy in Chinese patients. All data analyses were performed using R software (version 4.3.1).

## Results

### Clinical baseline characteristics of patients with PDAC

We included 10,813 patients with PDAC from the SEER database, among which 445 had LM. Meanwhile, 7,569 (70%) patients were assigned to the training cohort, and 3,244 (30%) patients were included in the internal validation cohort; there were no significant differences in the patient characteristics between these two cohorts (**Table 1**). The baseline clinical characteristics of the external validation group are shown in **Table 2**. A total of 132 patients with PDAC were included, 32 of whom developed LM.

**Table 1:** Baseline characteristics of PDAC patients over training cohort and internal validation cohort.

Characteristic	Training(N=7569)	Validation(N=3244)	Overall(N=10813)	P-value
Age, year				
<65	2906 (38.4%)	1246 (38.4%)	4152 (38.4%)	1
≥65	4663 (61.6%)	1998 (61.6%)	6661 (61.6%)	
Race				
Black	758 (10.0%)	332 (10.2%)	1090 (10.1%)	0.339
Other	638 (8.4%)	300 (9.2%)	938 (8.7%)	
White	6173 (81.6%)	2612 (80.5%)	8785 (81.2%)	
Sex				
Female	3649 (48.2%)	1595 (49.2%)	5244 (48.5%)	0.372
Male	3920 (51.8%)	1649 (50.8%)	5569 (51.5%)	
Grade				
I	861 (11.4%)	329 (10.1%)	1190 (11.0%)	0.258
II	3682 (48.6%)	1582 (48.8%)	5264 (48.7%)	
III	2949 (39.0%)	1302 (40.1%)	4251 (39.3%)	
IV	77 (1.0%)	31 (1.0%)	108 (1.0%)	
Tumour size, mm				
≤25	1955 (25.8%)	802 (24.7%)	2757 (25.5%)	0.286
26~40	3053 (40.3%)	1358 (41.9%)	4411 (40.8%)	
≥41	2561 (33.8%)	1084 (33.4%)	3645 (33.7%)	
Primary site				
Body/Tail	1714 (22.6%)	720 (22.2%)	2434 (22.5%)	0.104
Head	4894 (64.7%)	2155 (66.4%)	7049 (65.2%)	
Others	961 (12.7%)	369 (11.4%)	1330 (12.3%)	
AJCC T stage				
T1	373 (4.9%)	136 (4.2%)	509 (4.7%)	0.066
T2	1253 (16.6%)	496 (15.3%)	1749 (16.2%)	
T3	4911 (64.9%)	2133 (65.8%)	7044 (65.1%)	
T4	1032 (13.6%)	479 (14.8%)	1511 (14.0%)	
AJCC N stage				
N0	3412 (45.1%)	1407 (43.4%)	4819 (44.6%)	0.106
N1	4157 (54.9%)	1837 (56.6%)	5994 (55.4%)	
Lung metastasis				
No	7250 (95.8%)	3118 (96.1%)	10368 (95.9%)	0.459
Yes	319 (4.2%)	126 (3.9%)	445 (4.1%)	
Bone metastasis				
No	7480 (98.8%)	3203 (98.7%)	10683 (98.8%)	0.773
Yes	89 (1.2%)	41 (1.3%)	130 (1.2%)	
Liver metastasis				
No	6316 (83.4%)	2739 (84.4%)	9055 (83.7%)	0.213
Yes	1253 (16.6%)	505 (15.6%)	1758 (16.3%)	

**Table 2:** Baseline characteristics of PDAC patients over external validation cohort.

	Non-LM(N=100)	LM(N=32)	Overall(N=132)	P-value
Age, year				
<65	50 (50.0%)	14 (43.8%)	64 (48.5%)	0.680
≥65	50 (50.0%)	18 (56.3%)	68 (51.5%)	
Sex				
Female	69 (69.0%)	22 (68.8%)	91 (68.9%)	1
Male	31 (31.0%)	10 (31.3%)	41 (31.1%)	
Grade				
I	22 (22.0%)	2 (6.3%)	24 (18.2%)	<0.001
II	63 (63.0%)	6 (18.8%)	69 (52.3%)	
III	15 (15.0%)	21 (65.6%)	36 (27.3%)	
IV	0 (0%)	3 (9.4%)	3 (2.3%)	
Tumour size, mm				
≤25	30 (30.0%)	8 (25.0%)	38 (28.8%)	0.660
≥41	23 (23.0%)	6 (18.8%)	29 (22.0%)	
26~40	47 (47.0%)	18 (56.3%)	65 (49.2%)	
Primary site				
Pancreas other	9 (9.0%)	0 (0%)	9 (6.8%)	0.010
Pancreatic body	18 (18.0%)	7 (21.9%)	25 (18.9%)	
Pancreatic head	63 (63.0%)	15 (46.9%)	78 (59.1%)	
Pancreatic tail	10 (10.0%)	10 (31.3%)	20 (15.2%)	
AJCC T stage				
T1	17 (17.0%)	1 (3.1%)	18 (13.6%)	0.137
T2	61 (61.0%)	23 (71.9%)	84 (63.6%)	
T3	22 (22.0%)	8 (25.0%)	30 (22.7%)	
AJCC N stage				
N0	67 (67.0%)	10 (31.3%)	77 (58.3%)	<0.001
N1	31 (31.0%)	3 (9.4%)	34 (25.8%)	
N2	2 (2.0%)	19 (59.4%)	21 (15.9%)	
Bone metastasis				
No	98 (98.0%)	28 (87.5%)	126 (95.5%)	0.046
Yes	2 (2.0%)	4 (12.5%)	6 (4.5%)	
Liver metastasis				
No	88 (88.0%)	10 (31.3%)	98 (74.2%)	<0.001
Yes	12 (12.0%)	22 (68.8%)	34 (25.8%)	

### Independent risk factors for LM in PDAC

As shown in **Table 3**, we performed univariate logistic regression analysis on the ten latent factors and then six LM-related variables, including grade, tumour size, primary site, AJCC T stage, bone metastasis, and liver metastasis. These variables were then subjected to a multivariate logistic regression analysis. The results showed that the independent predictors of LM in PDAC were the primary site, tumour size, bone metastasis, and liver metastasis.

**Table 3:** Univariate and multivariate logistic regression analysis of risk factors for LM in PDAC.

Characteristic	Univariate		Multivariate	
	HR 95% CI	P-value	HR 95% CI	P-value
Age, year				
<65				
≥65	1.198 (1.015-1.419)	0.076		
Race				
Black				
Other	1.142(0.796-1.639)	0.544		
White	1.010(0.779-1.333)	0.952		
Sex				
Female				
Male	0.926(0.790-1.086)	0.428		
Grade				
I				
II	1.015(0.766-1.367)	0.932	0.773(0.576-1.054)	0.161
III	1.508(1.042-2.024)	0.018	0.878(0.654-1.197)	0.479
IV	0.801(0.251-1.939)	0.714	0.370(0.110-0.956)	0.122
Tumour size, mm				
≤25				
26~40	1.515(1.165-1.990)	0.010	1.245(0.921-1.705)	0.242
≥41	3.742(2.938-4.827)	<0.001	2.032(1.520-2.759)	<0.001
Primary site				
Body/Tail				
Head	0.270(0.226-0.323)	<0.001	0.518(0.426-0.629)	<0.001
Others	0.766(0.611-0.953)	0.048	0.768(0.606-0.969)	0.064
AJCC T stage				
T1				
T2	3.044(1.981-4.931)	<0.001	1.192(0.712-2.065)	0.586
T3	0.819(0.536-1.322)	0.466	0.644(0.389-1.108)	0.166
T4	2.611(1.686-4.254)	<0.001	1.029(0.608-1.800)	0.931
AJCC N stage				
N0				
N1	0.846(0.722-0.993)	0.085		
Bone metastasis				
No				
Yes	12.640(9.149-17.295)	<0.001	5.277(3.701-7.467)	<0.001
Liver metastasis				
No				
Yes	8.406(7.132-9.920)	<0.001	4.906(4.080-5.904)	<0.001

### Construction and validation of the diagnostic model

**Figure 2:**
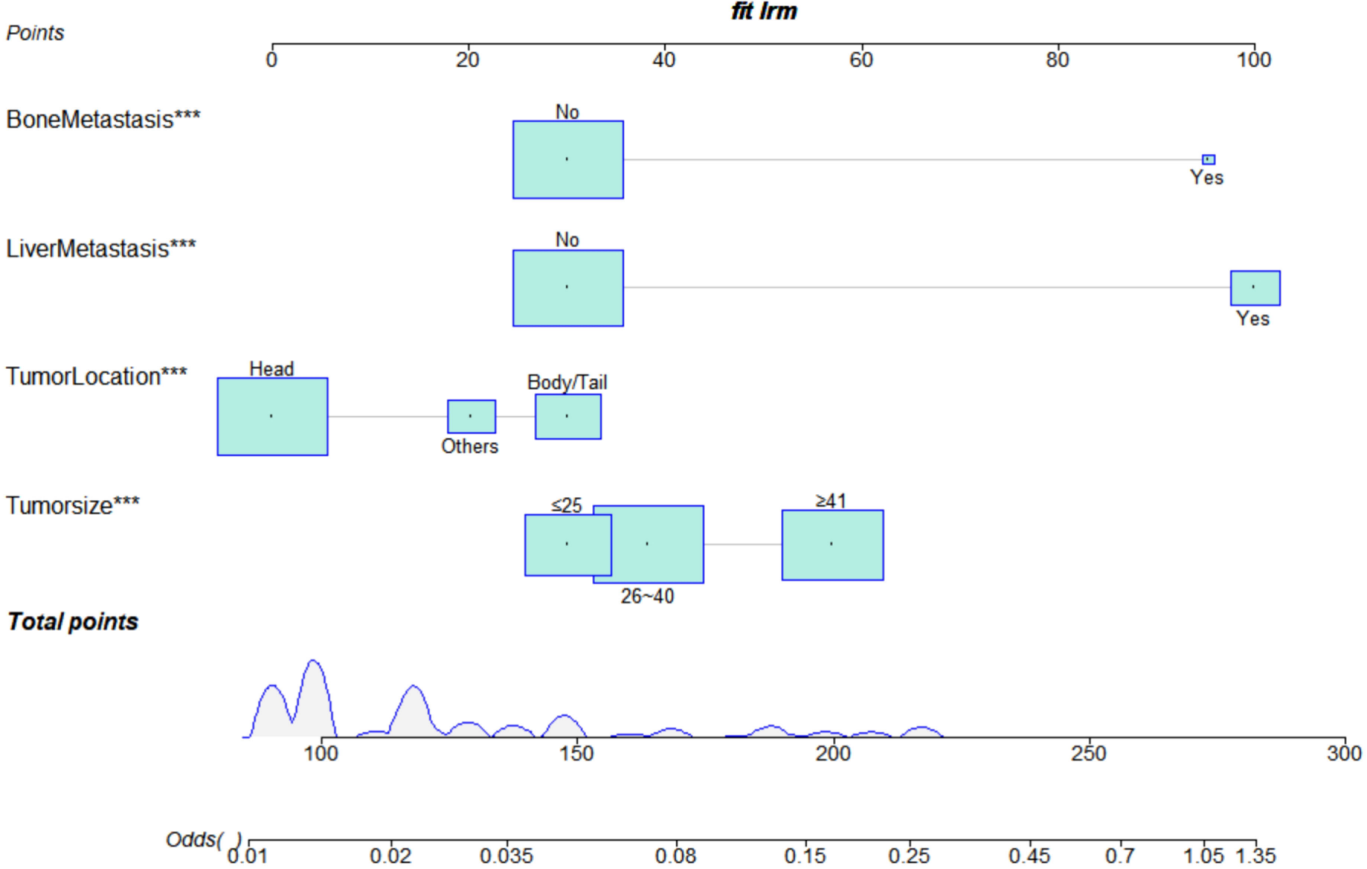
Nomogram for assessing the risk of lung metastasis in pancreatic ductal adenocarcinoma.

We constructed a risk prediction nomogram model for PCLM based on the primary site, tumour size, bone metastasis, and liver metastasis (**Figure 2**).

**Figure 3:**
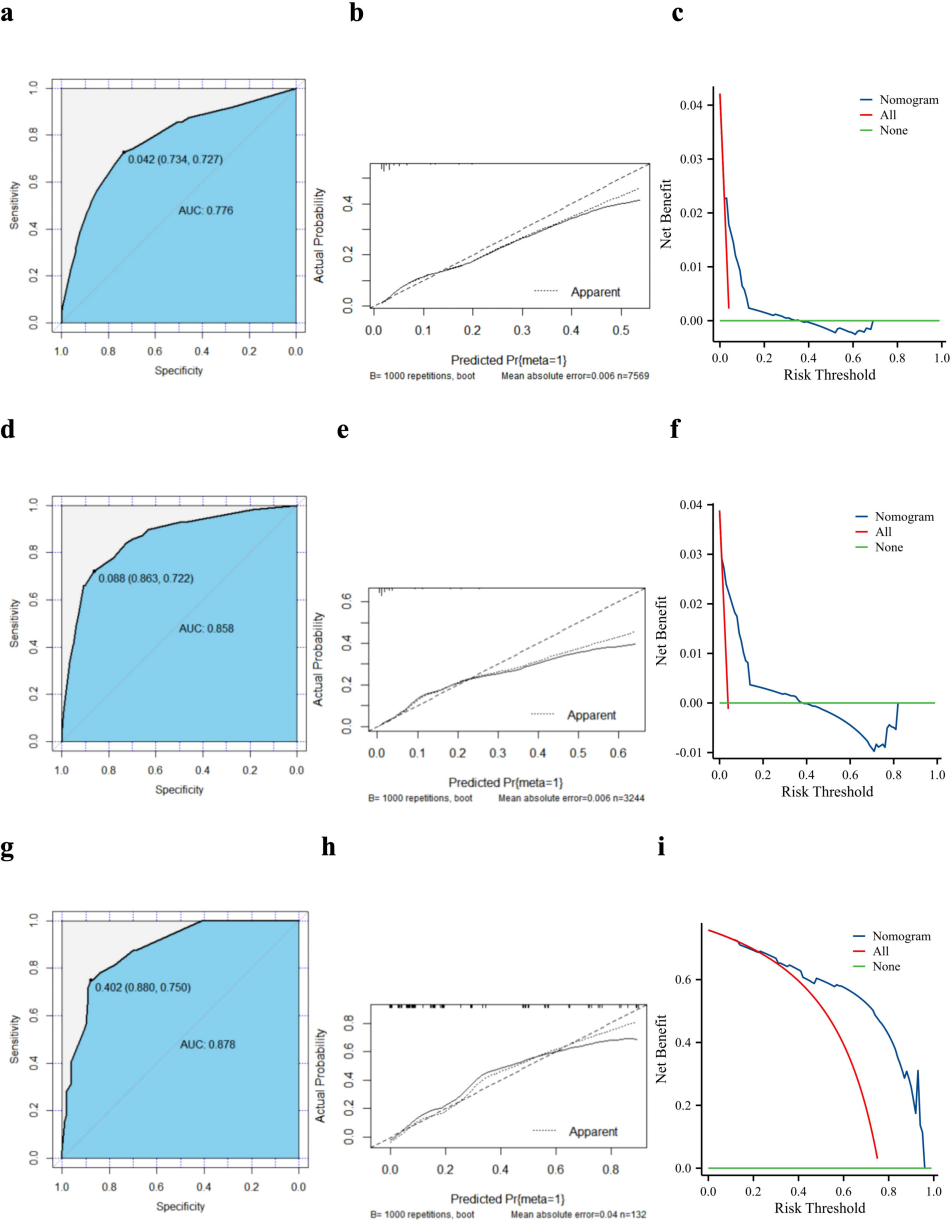
Validation of the diagnostic model. Receiver operating characteristic curves, calibration plots, and decision curve analysis of the nomogram for the risk of lung metastasis in pancreatic ductal adenocarcinoma based on the training cohort **(a–c)**, internal validation cohort **(d–f)**, and external validation cohort **(g–i)**. AUC, area under the curve.

A validation curve was constructed in accordance with the model shown in the nomogram. The ROC analysis showed that the area under the curve (AUC) value of the nomogram was 0.776 in the training cohort and 0.858 in the internal cohort, indicating a strong discriminative power of the model (**Figures 3a, d**). The calibration curves were highly consistent with the predictions (**Figures 3b, e**). The DCA indicated the high validity of the nomogram model in clinical practice (**Figures 3c, f**).

We created an external validation cohort to evaluate the model further using the same analysis. The ROC analysis showed that the AUC value of the nomogram was 0.878, which indicated that the model also had a good discriminatory ability in Chinese patients with PC (**Figure 3g**). The calibration curve showed good agreement between the nomogram predictions and actual observations, and the external validation cohort was almost identical to the training cohort (**Figure 3h**). Furthermore, the DCA showed clinical effectiveness similar to that in the training cohort (**Figure 3i**).

**Figure 4:**
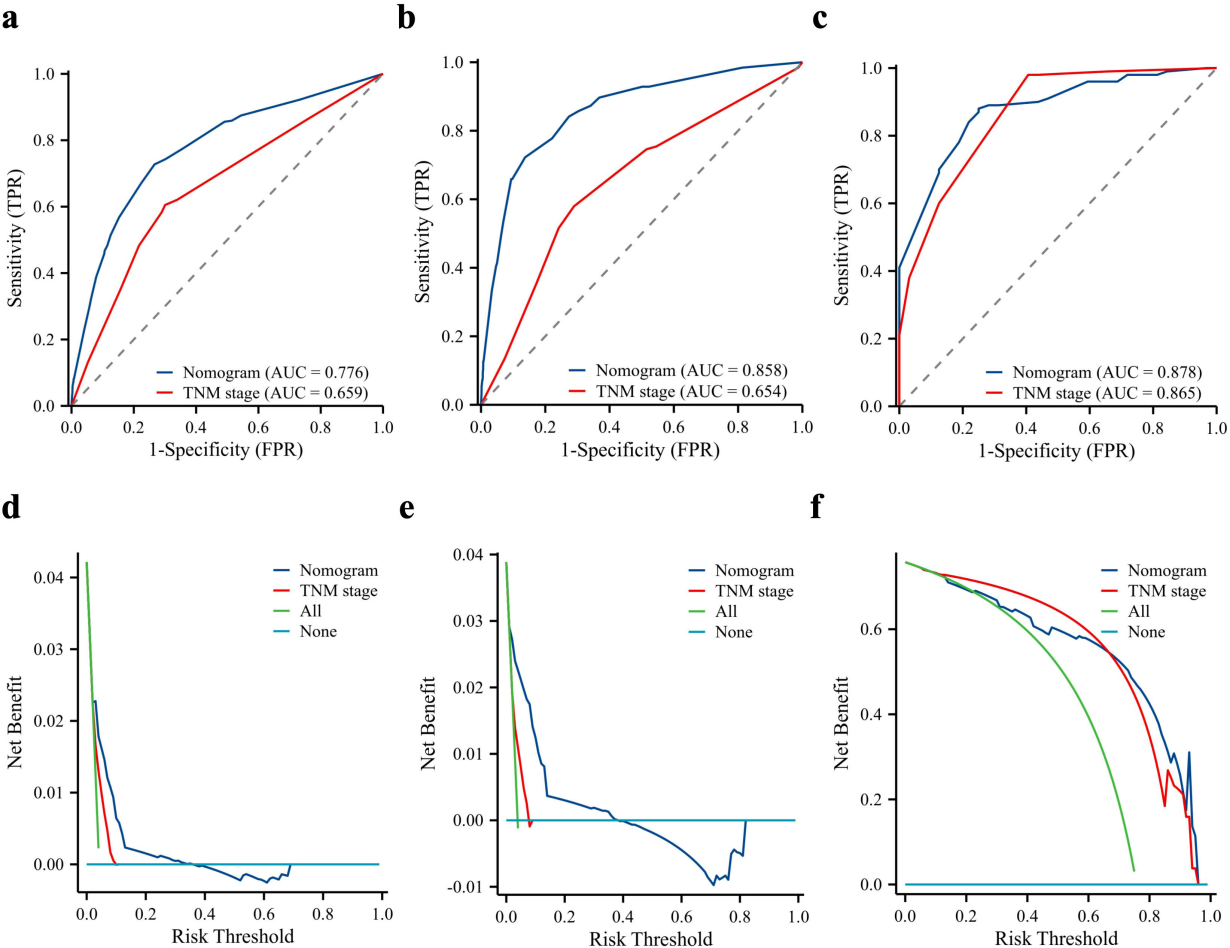
Predictive performance of the nomogram compared to the TNM stage. Comparison of receiver operating characteristic curve and decision curve analysis between nomogram and TNM stage in the training cohort **(a, d)**, internal validation cohort **(b, e)**, and external validation cohort **(c, f)**. AUC, area under the curve.

The TNM system is an internationally accepted criterion for cancer staging commonly used to predict the clinical behaviour of malignant tumours and guide clinical decision-making. We compared the predictive effects of both methods by plotting traditional TNM staging against the ROC curves of the nomogram and DCA. The analysis showed that the nomogram had better discrimination than TNM staging in both the modelled and external validation cohorts (**Figures 4a–f**).

### Clinical baseline characteristics of patients with PDAC and LM

A total of 445 patients with LM were used to identify the prognostic factors. All patients were randomly categorised at the ratio of 7:3 into training (n = 311) and validation (n = 134) cohorts. No variables were significantly different between the two cohorts (**Table 4**).

**Table 4:** Baseline characteristics of PDAC patients with LM among training cohort and internal validation cohort.

Characteristic	Training(N=311)	Validation(N=134)	Overall(N=445)	P-value
Age, year				
<65	110 (35.4%)	43 (32.1%)	153 (34.4%)	0.576
≥65	201 (64.6%)	91 (67.9%)	292 (65.6%)	
Race				
Black	35 (11.3%)	9 (6.7%)	44 (9.9%)	0.223
Other	27 (8.7%)	16 (11.9%)	43 (9.7%)	
White	249 (80.1%)	109 (81.3%)	358 (80.4%)	
Sex				
Female	161 (51.8%)	63 (47.0%)	224 (50.3%)	0.414
Male	150 (48.2%)	71 (53.0%)	221 (49.7%)	
Income				
<55,000	30 (9.6%)	8 (6.0%)	38 (8.5%)	0.277
≥55,000	281 (90.4%)	126 (94.0%)	407 (91.5%)	
Marital status				
Married	174 (55.9%)	82 (61.2%)	256 (57.5%)	0.231
Other	88 (28.3%)	39 (29.1%)	127 (28.5%)	
Unmarried	49 (15.8%)	13 (9.7%)	62 (13.9%)	
Grade				
I	27 (8.7%)	14 (10.4%)	41 (9.2%)	0.804
II	133 (42.8%)	51 (38.1%)	184 (41.3%)	
III	149 (47.9%)	68 (50.7%)	217 (48.8%)	
IV	2 (0.6%)	1 (0.7%)	3 (0.7%)	
Primary site				
Body/Tail	133 (42.8%)	64 (47.8%)	197 (44.3%)	0.550
Head	116 (37.3%)	48 (35.8%)	164 (36.9%)	
Others	62 (19.9%)	22 (16.4%)	84 (18.9%)	
Tumour size, mm				
≤25	37 (11.9%)	18 (13.4%)	55 (12.4%)	0.369
≥41	187 (60.1%)	71 (53.0%)	258 (58.0%)	
26~40	87 (28.0%)	45 (33.6%)	132 (29.7%)	
AJCC T stage				
T1	11 (3.5%)	4 (3.0%)	15 (3.4%)	0.663
T2	100 (32.2%)	48 (35.8%)	148 (33.3%)	
T3	125 (40.2%)	46 (34.3%)	171 (38.4%)	
T4	75 (24.1%)	36 (26.9%)	111 (24.9%)	
AJCC N stage				
N0	156 (50.2%)	60 (44.8%)	216 (48.5%)	0.348
N1	155 (49.8%)	74 (55.2%)	229 (51.5%)	
Delayed treatment, month				
>1	302 (97.1%)	128 (95.5%)	430 (96.6%)	0.574
≤1	9 (2.9%)	6 (4.5%)	15 (3.4%)	
Surgery				
No	285 (91.6%)	120 (89.6%)	405 (91.0%)	0.599
Yes	26 (8.4%)	14 (10.4%)	40 (9.0%)	
Radiotherapy				
No	282 (90.7%)	123 (91.8%)	405 (91.0%)	0.844
Yes	29 (9.3%)	11 (8.2%)	40 (9.0%)	
Chemotherapy				
No	116 (37.3%)	61 (45.5%)	177 (39.8%)	0.128
Yes	195 (62.7%)	73 (54.5%)	268 (60.2%)	
Bone metastasis				
No	275 (88.4%)	127 (94.8%)	402 (90.3%)	0.057
Yes	36 (11.6%)	7 (5.2%)	43 (9.7%)	
Liver metastasis				
No	131 (42.1%)	53 (39.6%)	184 (41.3%)	0.689
Yes	180 (57.9%)	81 (60.4%)	261 (58.7%)	

### Construction and validation of the prognostic model

Six variables were related to the prognosis of patients with PDAC and LM: race, primary site, surgery, chemotherapy, bone metastasis, and liver metastasis. Independent prognostic factors, including the primary site, liver metastasis, surgery, and chemotherapy, were identified using multivariate Cox regression analysis (**Table 5**).

**Table 5:** Univariate and multivariate COX regression analysis of prognostic factors for PDAC patients with LM.

	Univariate		Multivariate	
	HR 95% CI	P-value	HR 95% CI	P-value
Age, year				
<65				
≥65	1.201(0.986-1.463)	0.069		
Race				
Black				
Other	0.751(0.490-1.151)	0.189	0.957(0.620-1.478)	0.843
White	0.728(0.531-0.997)	0.048	0.899(0.653-1.238)	0.514
Sex				
Female				
Male	1.136(0.942-1.371)	0.181		
Income				
<55,000				
≥55,000	0.780(0.559-1.089)	0.145		
Marital status				
Married				
Other	1.151(0.929-1.426)	0.199		
Unmarried	1.281(0.967-1.697)	0.084		
Grade				
I				
II	0.997(0.710-1.400)	0.985		
III	1.245(0.891-1.740)	0.198		
IV	0.819(0.253-2.655)	0.740		
Primary site				
Body/Tail				
Head	0.750(0.608-0.924)	0.007	0.798(0.644-0.990)	0.040
Others	0.845(0.653-1.095)	0.203	0.706(0.540-0.922)	0.011
Tumour size, mm				
≤25				
≥41	1.321(0.986-1.768)	0.062		
26~40	1.082(0.789-1.484)	0.627		
AJCC T stage				
T1				
T2	1.156(0.679-1.968)	0.593		
T3	0.806(0.475-1.368)	0.424		
T4	1.086(0.633-1.863)	0.766		
AJCC N stage				
N0				
N1	1.015(0.842-1.225)	0.874		
Delayed treatment, month				
>1				
≤1	0.963(0.564-1.645)	0.891		
Surgery				
No				
Yes	0.464(0.333-0.678)	<0.001	0.506(0.360-0.713)	<0.001
Radiotherapy				
No				
Yes	1.002(0.720-1.394)	0.990		
Chemotherapy				
No				
Yes	0.377(0.277-0.411)	<0.001	0.319(0.260-0.392)	<0.001
Bone metastasis				
No				
Yes	1.389(1.009-1.912)	0.044	1.316(0.953-1.819)	0.096
Liver metastasis				
No				
Yes	1.843(1.519-2.238)	<0.001	1.517(1.239-1.857)	<0.001

**Figure 5:**
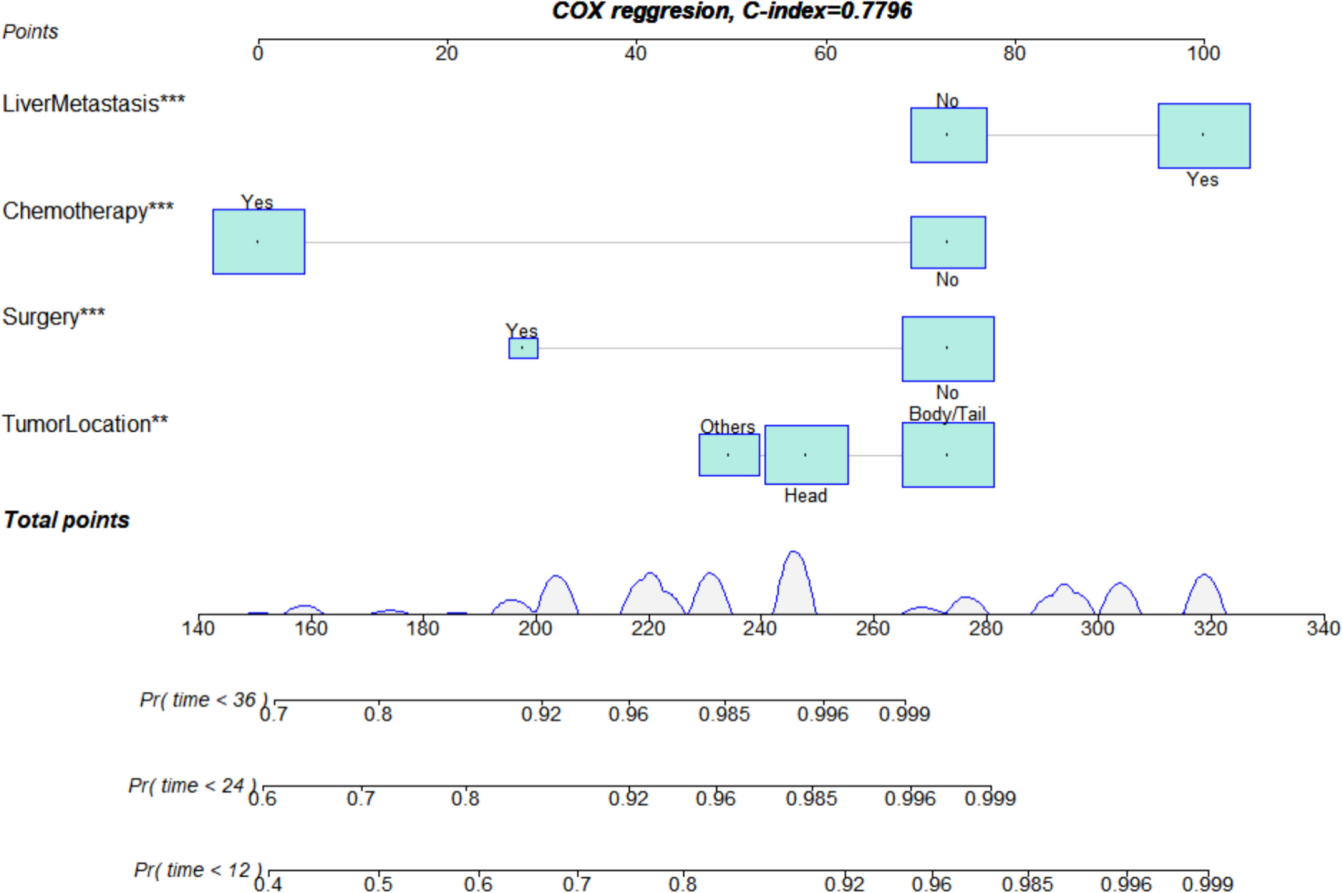
Nomogram for assessing the prognosis of lung metastasis in pancreatic ductal adenocarcinoma. **p<0.01, ***p<0.001.

We constructed a prognosis prediction nomogram model for PDAC with LM based on the primary site, liver metastasis, surgery, and chemotherapy (**Figure 5**).

**Figure 6:**
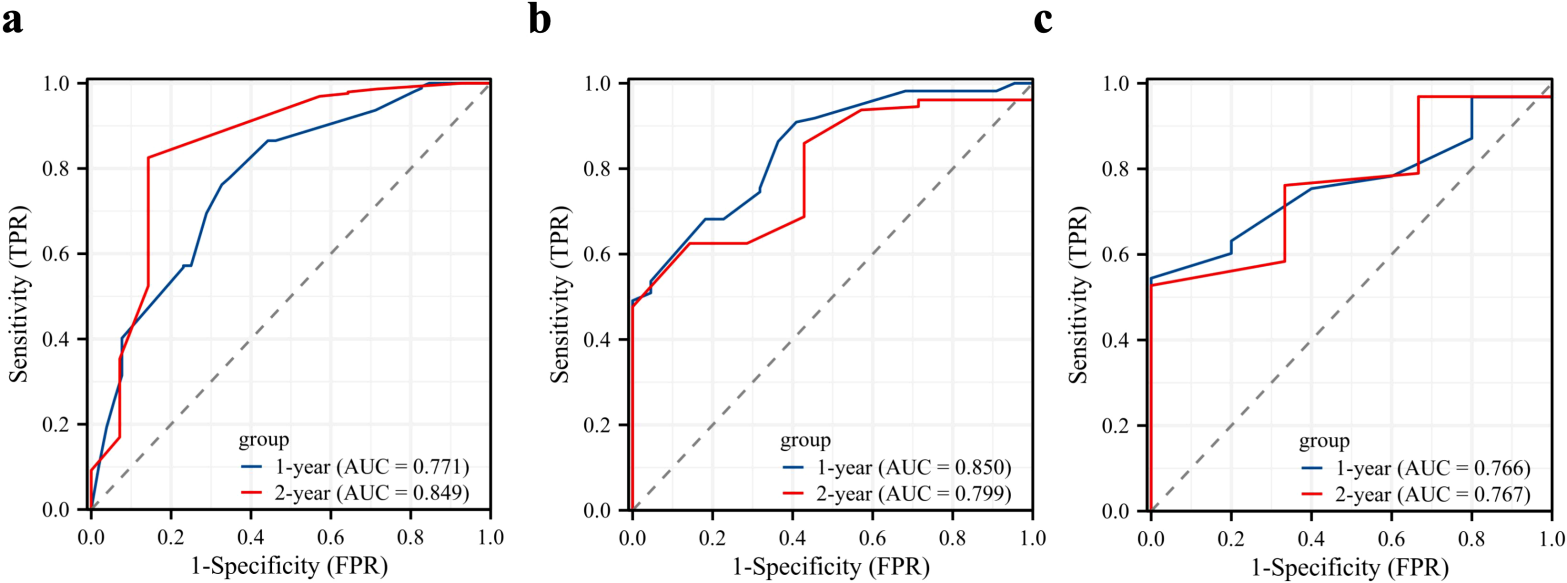
Validation of the prognostic model. Receiver operating characteristic curves for the prognostic nomogram in predicting the 1- and 2-year overall survival in the training cohort **(a)**, internal validation cohort **(b)**, and external validation cohort **(c)**.

**Figure 7:**
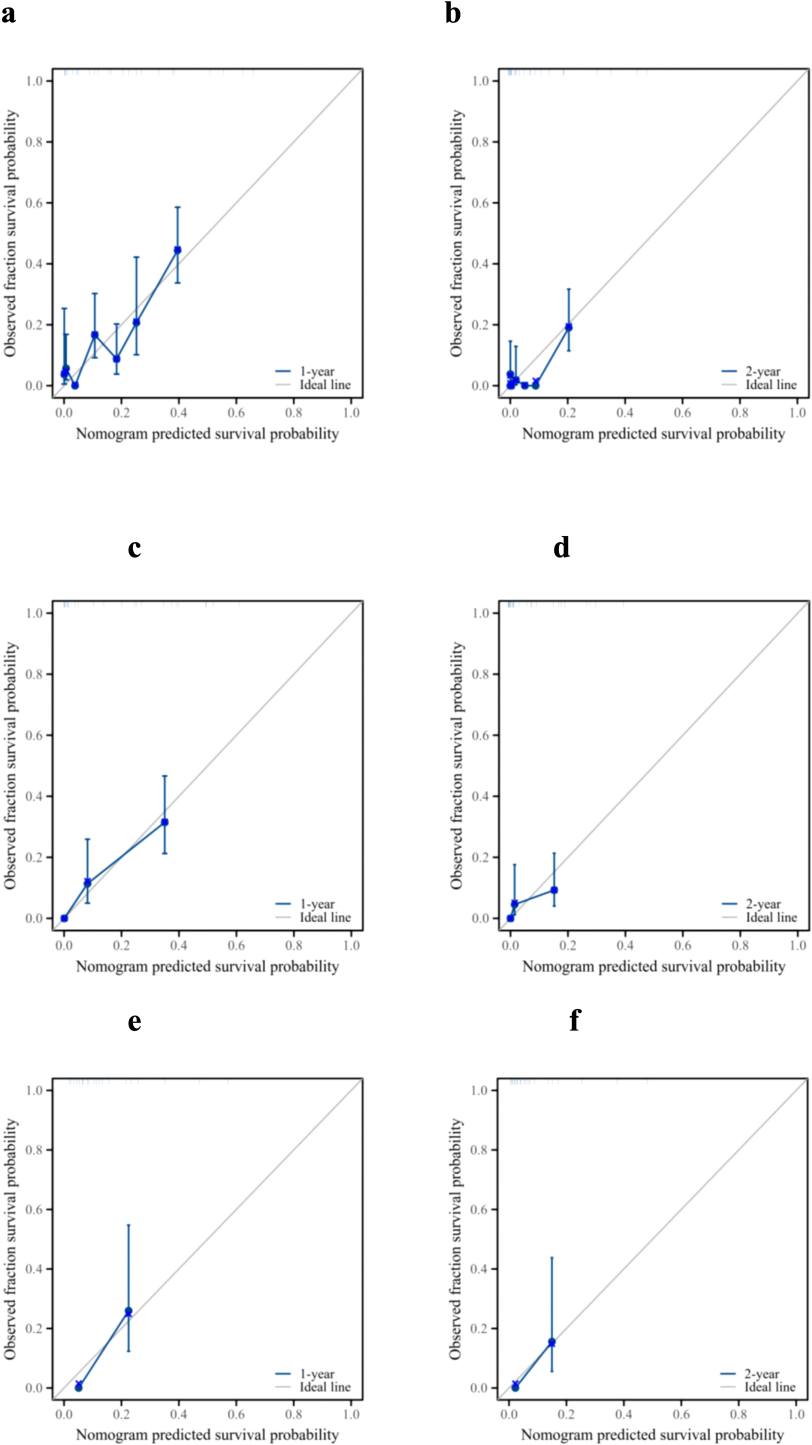
Prognostic performance of the model. Calibration curves for the 1- and 2-year overall survival of patients with pancreatic ductal adenocarcinoma and lung metastasis in the training cohort **(a, b)**, internal validation cohort **(c, d)**, and external validation cohort **(e, f)**.

The ROC analysis of the prognostic nomogram showed that the AUC values for 1-year and 2-year OS in the training cohort were 0.771 and 0.849, respectively (**Figure 6a**). We performed ROC analysis using internal and external validation cohorts to validate the nomogram further. The AUC values in the internal and external validation cohort for 1-year and 2-year OS were 0.850 and 0.799 (**Figure 6b**) and 0.766 and 0.767 (**Figure 6c**), respectively. Thus, the prognosis nomogram performed better in predicting OS at 1 and 2 years. The calibration curves of the prognostic nomogram also showed strong agreement between the predicted OS and the actual values (**Figure 7**).

**Figure 8:**
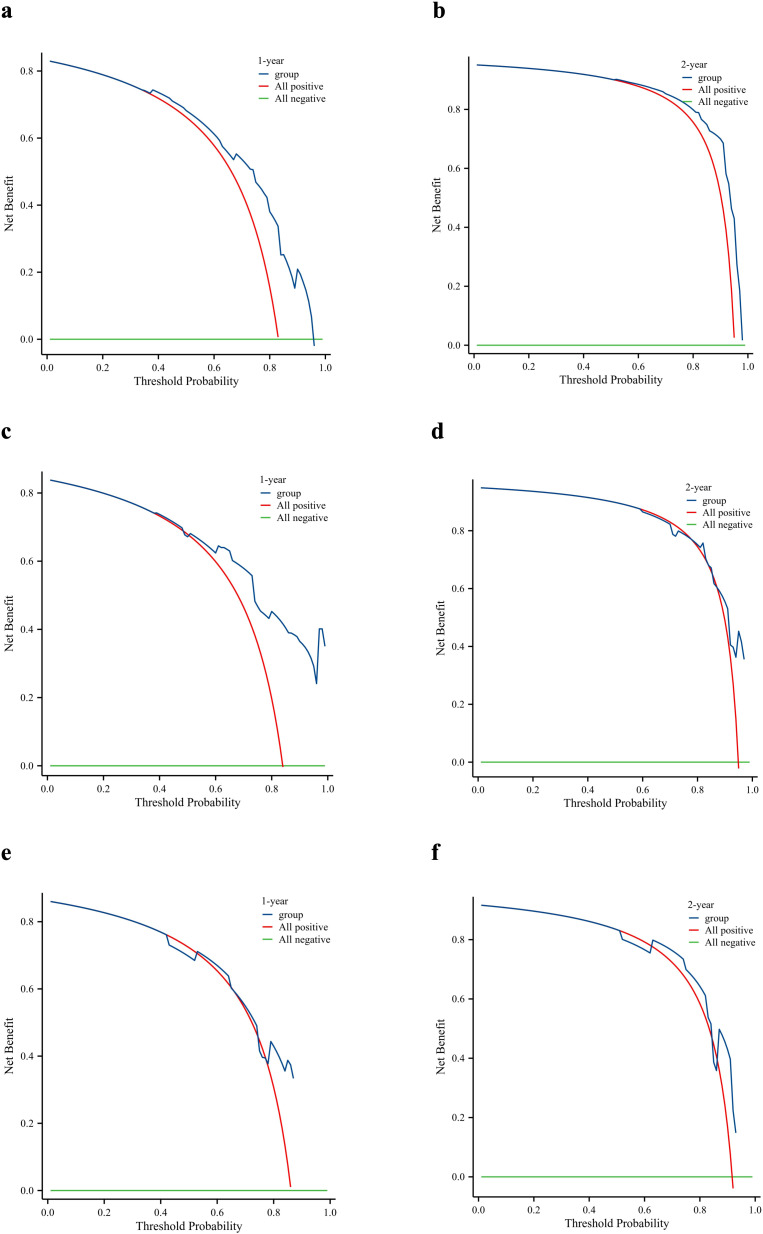
Clinical utility of the nomogram. Decision curve analysis of the 1- and 2-year survival benefits in the training cohort **(a, b)**, internal validation cohort **(c, d)**, and external validation cohort **(e, f)**.

DCA was used to evaluate the clinical value of the nomogram. As shown in **Figure 8**, the prognostic nomogram showed significant positive gains when predicting the 1- and 2-year mortality risks of patients in the training cohort (**Figures 8a, b**). The 1-year mortality risk of the patients in the internal validation cohort also showed remarkable positive gains (**Figure 8c**). Furthermore, the nomogram showed positive gains within a range when predicting the 2-year mortality risk of patients in the internal validation cohort and the 1- and 2-year mortality risks of patients in the external validation cohort (**Figure 8**). Therefore, the nomogram has important clinical utility in predicting the OS of patients with PDAC and LM.

**Figure 9:**
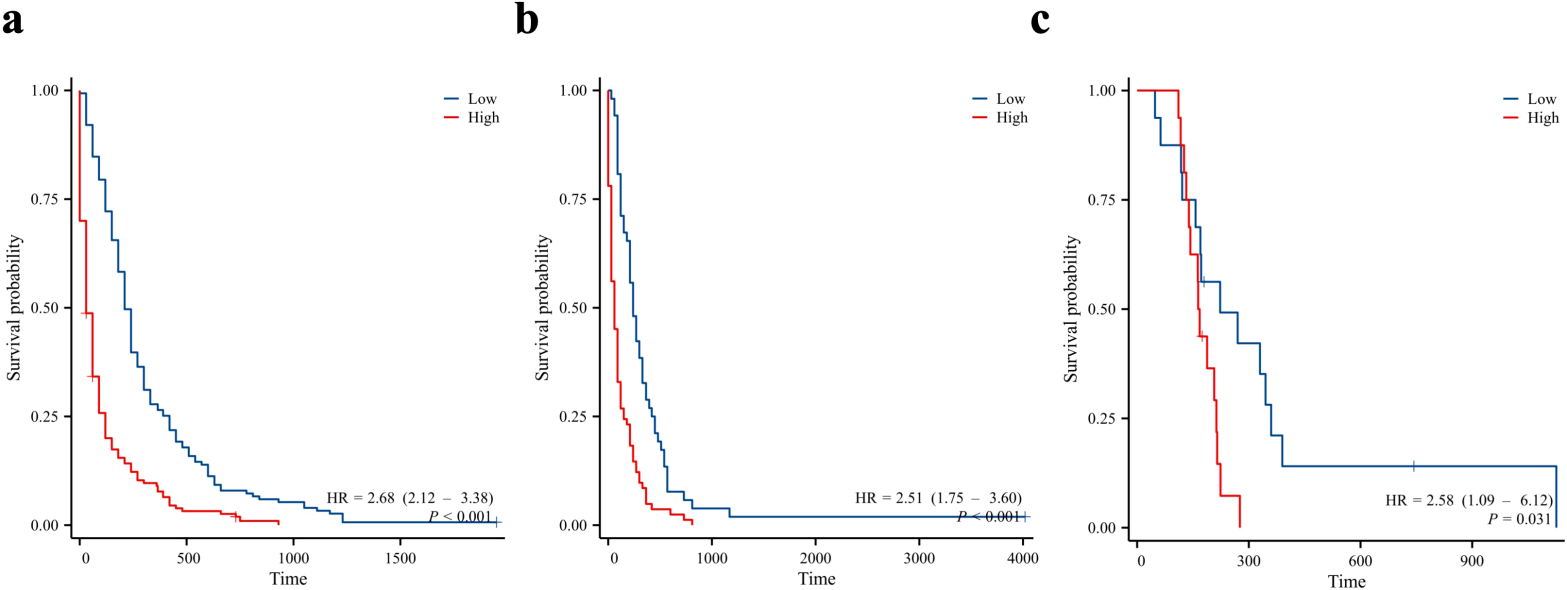
Kaplan-Meier curves for overall survival in patients in the low and high-risk groups. Training cohort **(a)**, internal validation cohort **(b)**, and external validation cohort **(c)**.

Finally, we divided the patients into the training, internal validation, and external validation cohorts into high- and low-risk groups according to the median risk score. Kaplan–Meier survival curves showed that all high-risk groups in these three cohorts had significantly lower prognoses than the low-risk groups (**Figure 9**).

## Discussion

PDAC is the leading cause of cancer-related death worldwide with an extremely lower 5-year overall survival (32). Since most patients present atypical symptoms at early stage (33), they are often diagnosed with locally advanced (30–35%) or metastatic (50–55%) disease (5). The lung is the second most common site of metastasis, besides the liver, with the incidence of LM reported to be 4.76–12.3% (6, 7). Most patients with PDAC and LM cannot undergo surgical treatment, which greatly increases mortality. Unlike primary lung cancer, PDAC with LM showed no obvious symptoms, such as hemoptysis or cough. Therefore, timely diagnosis of LM in patients with PDAC is particularly important. In the present study, models were established using the SEER database and validated using our single-center data to help clinical diagnosis and treatment.

Our research has revealed that primary tumours in the pancreatic body and tail are more prone to develop into liver metastases (LM) compared with tumours in the pancreatic head. This may be attributed to the fact that they are frequently detected at a late stage due to the absence of obstructive jaundice. Moreover, tumours in the pancreatic body or tail are generally larger at the time of initial diagnosis and more susceptible to metastases (34). Consequently, these patients are at a higher risk of pulmonary metastasis, which is in line with the results of previous studies. Patients with larger tumours at the primary site are more likely to develop distant metastases. In combination with the findings of previous studies, this conclusion might only be applicable to patients in the early stages of pancreatic cancer (PC). When PC exhibits local or distant spread, it is not suitable for predicting lung metastasis; however, at this stage, the tumour size is large and the potential for lung metastasis is increased (35). Among the data extracted from the SEER database, 13.46% of patients with liver metastasis had LM, and 29.94% of those with bone metastases had LM. This finding indicates a high probability of LM in the presence of multiple distant metastases.

Surgery represents the sole curative treatment for pancreatic ductal adenocarcinoma (PDAC). Nevertheless, the PDAC diagnosis and treatment guidelines do not advocate surgical resection in patients with distant metastases. Chemotherapy is routinely carried out following the radical resection of PDAC, and the separate application of gemcitabine or fluorouracil is recommended (19). However, PDAC exhibits certain resistance to chemotherapeutic drugs, particularly gemcitabine. Studies have demonstrated that PDAC cells are more resistant to gemcitabine compared with other chemotherapeutic drugs (20, 21). This significantly restricts the use of gemcitabine and deteriorates the patient’s prognosis. Consequently, there is an urgent necessity for practical tools and concise clinical treatment options. In this respect, identifying the prognostic factors for patients with PDAC and liver metastases (LM) and developing a new tool for clinicians could enable personalised treatment options for each patient.

This study revealed that the primary site, liver metastasis, surgery, and chemotherapy were independent prognostic factors for patients with pancreatic ductal adenocarcinoma (PDAC) and liver metastasis (LM). The median overall survival (OS) of patients with isolated liver metastasis (IL) was merely 561 days (9). Early diagnosis and prompt treatment can enhance the prognosis of patients. As in previous studies (36–38), we found that the prognosis of patients who underwent surgery and chemotherapy was significantly improved. Additionally, in patients who are unable to undergo surgery, combining gemcitabine with nab paclitaxel (GnP) can substantially improve the feasibility and efficacy of surgery (39). Liver metastasis is the predominant site of distant metastasis in PDAC; thus, patients with PDAC and LM often have liver metastasis concurrently, and the prognosis of patients with multiple distant metastases is undoubtedly very poor (6). We also found that the prognosis of primary tumours in the pancreatic head was far superior to that of tumours in other sites. This might be related to the tendency of patients with pancreatic head cancer to develop jaundice during early diagnosis and treatment. It has also been demonstrated that PDACs located in the pancreatic body and tail are rich in genetic programs involved in tumour invasion and epithelial to mesenchymal transition, as well as having a poor antitumour immune response, resulting in a better prognosis of head PDAC than body and tail PDACs (40, 41).

Predictive and prognostic nomograms were constructed based on these factors. The ROC AUCs of the internal validation and external validation cohorts were high. Meanwhile, the calibration plots and DCA of the internal and external validation cohorts showed good discriminative power and clinical applicability. Therefore, these two nomograms can predict the risk and prognosis of LM in PDAC and help make individualised clinical decisions. The two prediction models derived from this study will become convenient and effective practical tools in clinical practice.

This study has some limitations. Although external validation was performed in this study, the amount of data in the external validation cohort was small; therefore, the results were not convincing. Next, neither the internal validation data from the SEER database nor the external data considered potential factors that may influence the diagnosis, such as body mass index, alcohol consumption, smoking, tumour biomarkers, and routine blood tests. Furthermore, we can add genetic testing as one of the predictive factors for diagnosis and prognosis on the basis of the clinical characteristics to enhance the reliability of the prediction (42). Despite these limitations, the nomogram remains practical, and we expect that prediction model validation will be further improved in the near future.

## Conclusion

In summary, we identified risk and prognostic factors for PDAC with LM based on univariate, multivariate logistic, and Cox regression analyses. Risk factors include the primary site, tumour size, bone metastasis, and liver metastasis. Prognostic factors included the primary site, liver metastasis, surgery, and chemotherapy. We established two nomograms for the diagnosis and prognosis of PDAC with LM using R software, which can help clinicians effectively identify high-risk patients and target them individually.

## Data Availability

The raw data supporting the conclusions of this article will be made available by the authors, without undue reservation.
